# Supplementary material for: Spatiotemporal dynamics of self-organized branching in pancreas-derived organoids
Source: Nat Commun. 2022 Sep 5;13:5219. doi: 10.1038/s41467-022-32806-y (PMC9445099; doi:10.1038/s41467-022-32806-y)
Supplement: Supplementary file 1 — Supplementary Information [file 41467_2022_32806_MOESM1_ESM.pdf]

Supplementary Materials for

Spatiotemporal dynamics of self-organized branching in pancreas-derived organoids

S. Randriamanantsoa, A. Papargyriou, H. C. Maurer, K. Peschke, M. Schuster, G. Zecchin, K. Steiger, R. Öllinger, D. Saur,

C. Scheel, R. Rad, E. Hannezo, M. Reichert*, A.R. Bausch*

These authors contributed equally: S. Randriamanantsoa, A. Papargyriou

*Correspondence to: abausch@mytum.de, maximilian.reichert@tum.de

This PDF file includes:

Supplementary Note

Figs. S1 to S7

Tables S1 to S3

References

Other Supplementary Materials for this manuscript include the following:

Movies S1 to S15

Supplementary Note

I. MINIMAL ANALYTICAL MODEL OF BRANCHING ORGANOID GROWTH

We first provide detail for the minimal theoretical model used to understand the branching structure of pancreatic organoids. We start with the case of a single branch, before moving to the description, backed by numerical simulations, of entire branched trees.

A. Single branch elongating without branching, nor proliferative feedback

We consider a cylindrical branch with width w (or radius $r = w/2$) and length L, made up of N cells with volume V_c, so that by volume conservation, branch width and length must be related by $\pi L w^2/4 = NV_c$. We consider that cells divide at a constant rate k_d, and that the cell at the very tip exerts a pulling force f_0 on the tube, which tends to elongate it. We denote as ζ the friction coefficient of cells migrating through the matrix.

Force balance on the tip cell then reads:

$$\zeta L'(t) = f_0 + \sigma_{branch} \qquad \text{(S1)}$$

i.e. the tube can elongate at speed $v = L'(t)$ either due to active migration at the tip (which predicts a linear increase at speed f_0/ζ), or due to compressive forces σ_{branch} from the bulk of the follower cells in the branch which can be dividing. A simple assumption is that those compressive forces are dependent on the 1D cell density $\rho(t) = N(t)/L(t)$, so that $\sigma_{branch} = \chi\rho(t)$ where χ is a compressibility [1]. We now write the evolution of cell numbers in the branch, which can increase due to proliferation, or decrease due to elongation:

$$N'(t) = L'(t)\rho(t) + L(t)\rho'(t) = k_d N(t) \qquad \text{(S2)}$$

so that force balance can be re-wrote as

$$\zeta L'(t) = f_0 + \chi e^{k_d t}/L(t) \quad \text{(S3)}$$

In this simplified model, there is no feedback on cellular proliferation, so that the cell number increases exponentially, as does length $L(t) \propto e^{k_d t/2}$ at long time scales ($L \rightarrow\infty$). On the other hand, migration force f_0 causes a linear increase, which is thus always negligible compared to proliferation at long enough time scales. Moreover, in the early time points, one expects the branch to be under tension (because $f_0 \gg \sigma_{branch}$, i.e. tips pull more than they are pushed) - which is the time at which there could be breakage as observed in the data (see for instance the "high branch count" real organoid in Fig. 4h, where some of the cells at the tips occasionally transiently detach). In the late time points, proliferation always wins and catches up, so that the length increases exponentially, pushed by the back. Note however that even in this simple scenario, the cell density also exponentially increases (because $N(t) \propto e^{k_d t}$, so much faster than length), which is due to frictional slowing down.

B. Single branch elongating without branching, but with proliferative feedback

This is clearly unphysical, and feedback mechanisms on proliferation must be included in the theory. Indeed, there is extensive evidence for stress/density feedbacks on proliferation [2–5], which has been proposed as a source of stabilization of tissue growth [6,7]. At linear order, the conservation equation for cell numbers with feedback reads:

$$N'(t) = L'(t)\rho(t) + L(t)\rho'(t) = k_d N(t)(1 - \alpha\rho(t)) \quad \text{(S4)}$$

Note that because we have assumed a simple linear relationship between stress and density, both enter the feedback in the same way, making this equation quite general [6,7]. This regulates density to a well-defined maximal value of $\rho_0 = 1/\alpha$. Under this assumption, density will always saturate to ρ_0, while length grows linearly with slope $L'(t) = (f_0 + \chi\rho_0)/\zeta$. This means that a phase of exponential growth (proliferation-limited) will be succeeded by a phase of linear

growth (migration-limited), due to the frictional forces penalizing growth-driven motion (note that for the limit $\zeta \rightarrow 0$, we get back to the results of the first section as expected).

Importantly, as long as the maximal proliferative stress $\chi\rho_0$ is larger than migration forces f_0, the branch will always transition from early tension/low density (where migration forces thin the branch more than it can be replenished by proliferation), to a state of late compression/high density (where proliferation catches up).

C. Branching tree growth with constant tip speed and no proliferative feedback

This analysis suggests that growth of a single branch will always converge to a linear regime in the presence of proliferative feedbacks. However, we haven't considered so far the branching of one tip into two (rate k_b), which drives branching morphogenesis. For tip branching, the total number of tips grows in time as $T(t) \propto e^{k_b t}$, with the total number of branches scaling as $2T(t)$ (for a symmetrically branching tree). If all tips grow with speed v_0, this means that the total length of the tree (equal to the sum of all branches) will scale as

$$L(t) = \frac{v_0}{k_b} e^{k_b t} \quad \text{(S5)}$$

and if cellular proliferation occurs at a constant rate k_d, the 1D cellular density (or branch radius) will scale as

$$\rho(t) = \frac{k_b}{v_0} e^{(k_d - k_b)t} \quad \text{(S6)}$$

which now compares two exponential processes, instead of a linear edge growth vs. an exponential bulk growth. In particular, if $k_b > k_d$, branching is too fast relative to cell division, and cellular density/branch thickness is expected to decrease. On the other hand, even if branching is slower than cell division, the transition towards significant branch thickening is expected to be strongly delayed compared to the previous section.

D. *Branching tree growth with constant tip speed and feedbacks*

Finally, combining the results from the previous sections, we can investigate the case of branching trees together with feedback on proliferation from branch thickness/density. Again, we make the simplifying assumption that all branches have equal thickness, something we will relax in numerical simulations in the next sections. The equations for total cell number $N(t)$, total tree length $L(t)$ and branch radius/width $r(t) \propto \sqrt{N(t)/L(t)}$ read:

$$\begin{cases} N'(t) = L'(t)r^2(t) + 2r(t)L(t)r'(t) = k_d N(t)(1 - \frac{w(t)}{w_0}) \\ L'(t) = \frac{v_0}{k_b} e^{k_b t} \end{cases} \quad \text{(S7)}$$

Interestingly, this again revealed two regimes of growth. At early stages of small cell numbers, migration dominates over proliferation, and branches are expected to be thin ($r \ll w_0$) allowing for fast growth at rates close to k_d. Thus, the total number of cells in a branched organoid is expected to grow exponentially as $e^{k_d t}$ at early time points. At later times however, the width converges towards a steady state, and we find that this growth phase is limited by the branching rate, with cell numbers growing as $e^{k_b t}$, so that

$$w(\infty) = w_0(1 - k_b/k_d) \quad \text{(S8)}$$

which corresponds to a width smaller than the maximal one w_0 at which proliferation stops, and at which the proliferation is slowed down enough to allow a steady-state, corresponding to a match between the branching rate k_b and the division rate $k_d(1 - w/w_0)$. It should be noted that this can only occur for $k_b < k_d$, which is similar to the criterion to the section above: if branching is faster than divisions, then branches would thin to zero-radius because branch proliferation could not keep up with the generation of moving tips.

II. PARAMETER ESTIMATION AND MODEL PREDICTIONS

We now seek to estimate each of the model parameter from the data, as well as experimentally verify some of our key assumptions.

We note that we intentionally restrict our model to the modelling of the branching and early thickening dynamics, occurring in general between day 1 and 9, and excluding the Lumen Formation phase of the organoids. Indeed, we experimentally find that, while the Onset, Extension, and early Thickening phases rely on similar processes of extension, branching, and proliferation, the late Thickening phase involves strong contraction, and the Lumen Formation phase appears to involve different processes, such as fluid intake, fluid pressure, or cell shape changes.

The formation of a lumen inside the branches also complicates the extension of the present model to the Lumen Formation phase: indeed, it would imply redefining the way some parameters are defined between phases, ultimately leading to a different model, with different validating experiments. For instance, the present manuscript uses the width of the branches as a proxy to estimate the maximal division rate k_d: this approximation is valid when the branches are not hollow; it however loses its validity when the lumen start swelling, and when fluid intake starts contributing to the thickness of the branch, independently of whether proliferation is actually taking place.

A. Assumptions of migratory and proliferation feedback

A key assumption that we have made is to i) assume a feedback from branch thickness on proliferation and ii) to take it as a linear dependency for simplicity (although the analysis would be largely similar in the presence of more complex functional forms). To check this in the data, we made use of a live-imaging dataset for organoid morphogenesis between day 7 to day 10, where we tracked the morphometrics of single branches in real-time for a few hours. This included a systematic quantification of tip speed, branch length and branch width for a number of randomly selected terminal branches in the organoid.

As a proxy for proliferation, we calculated at each time point the volume of a branch $V(t) = \pi w^2(t)L(t)/4$, and estimated the rate of volumetric growth $K(t)$ (which is a proxy for division rate k_d, although in principle a death rate of cells would negatively impact volume, so that the rate k_d could be seen as a balance between growth and loss) by comparing consecutive time points (separated by Δt) as $K(t) = \frac{V(t+\Delta t)-V(t)}{V(t)\Delta t}$. We checked that the average $< K(t) >$ over all branches was approximately constant in time, as expected when considering time scales of a few hours, which served as a control that the system is not slowing down due to photo-toxicity while imaging for instance.

Importantly, although $K(t)$ was very broadly distributed, plotting its value for each branch at each time point against the corresponding branch width $w(t)$ revealed a clear trend, where growth was markedly slowed down for larger branch radius, in a roughly linear manner. This verifies one of our key assumptions and allows us, from comparing data with the model of $K = k_d(1 - w/w_0)$, to fit both the maximal division rate k_d and the maximal width w_0 (at which proliferation is fully abrogated). Quantitatively, from a linear fit (Fig. 4b), we find $k_d = 3.9 \pm 1.2$ d^{-1} and $w_0 = 25 \pm 6$ μm (best fit parameter ± standard error of the fit).

A second important assumption from the section above is that we considered tip speed v_0 to be constant. Given the dependency of volumetric branch growth on branch width, we reasoned that it was an important control to see whether or not the speed of a given tip was correlated to the morphometric of its branch (width or length). However, we found no such correlation, and a constant value of $v_0 = 80$ μm/day was extracted from this dataset (Fig. 5h). We also found that detached cells migrated through the matrix at a similar velocity, arguing that tip movement/matrix degradation is the dominant phenomena setting tip growth (as opposed to stresses from the bulk/leader cells, which could explain why tip speed is not dependent on branch morphometrics).

B. *Estimation of other parameters*

Although measuring the branching rate from live-imaging is technically difficult, it can be estimated by comparing the tip elongation rate v_0 to the average length of a branch l_0, which we found to be $l_0 \approx 150\ \mu m$. From this, we could estimate that a constant branching rate of around once every 2 days $k_b \approx 0.55\ d^{-1}$, further corroborated by measuring the average number of branches of organoids from day 1 to day 9 (Fig. 4f, and see A. Summary of parameter estimation and simulation inputs in the Supplementary Note).

C. *Predictions from the model on unperturbed and perturbed organoid growth*

With these orders of magnitude, one can estimate that the transition from 1D cell density/branch thickness decrease (due to linear growth of the tip) to density/thickness increase where contact inhibition (due to proliferation) should occur on very rapid time scales of less than a day. This means that organoid growth should largely be over during these timescale (i.e. if there weren't branching events). However, as mentioned above, tip branching at rate k_b causes an exponential increase of the number of tips at rate $e^{k_b t}$, and if each of these tips elongate at a speed v_0, this means that the "edge" of the pancreas grows exponentially - rather than linearly in the case of a single branch (or classical 1D/2D/3D cohesive cellular colonies). We predict that such an exponential branching allows pancreatic organoids at their "full" exponential capability k_d to grow for longer (3-4 days), with contact inhibition occurring only after, and branching allowing continued exponential growth (albeit at a slow rate k_b), which matches well the experimental observations.

More quantitatively, turning to the prediction for the average number of cells in time in an organoid, we found that the model predicted very well the data across all time points (see Fig.

4d, even though the width to proliferation feedback was fitting only from short-term tracking around day 7). In particular, the model reproduces well the inflexion of growth shown in the data (Fig. 2d, Fig. 4d), which is predicted to arise due to the increase of average branch thickness in time (which feedbacks negatively on proliferation). Again, both of these predicted features agreed well with the data showing i) a steady decrease in the number of proliferative cells (as assessed by the Ki67 to DAPI ratio, Fig. 2c-e), and ii) a steady increase in branch thickness within the first days, then a more gradual plateau until day 10 (Fig. 4d).

We note that the apparent deviation of the "experimental" terminal branch average thickness from the "theoretical" value at day 8 and 9 (Fig. 4e), can be explained by the fact that, due to heterogeneity, some organoids phenotypes can display budding and occasionally start lumen expansion earlier than others, thereby driving the increase in apparent average thickness. As the current model does not take into account the lumen formation phase, for aforementioned reasons, such discrepancies might emerge. While, for comparison purposes with the model, we have taken care to exclude branches that displayed obvious, large and optically resolvable lumens, early fluid intake in branch tips (which can still lead to a noticeable increase in thickness), can be difficult to detect using the method described in the Methods section "Branch thickness measurement (static)".

We then proceeded to test some of the model predictions upon pharmacological perturbation. We first performed a batimastat treatment, which is expected to decrease the ability of tip cells to migrate through the ECM. Indeed, we found that upon addition of batimastat at day 7, average tip speed was reduced to zero. We input this in the model (together with a reduction of branch growth of 80% which we found experimentally, and could be due to secondary effects of the drug). We found that whereas WT organoids grew little in width between day 7 and day 9 (as expected in the second phase of growth predicted by the model), batimastat-treated organoids

were predicted to show larger branch thickness growth (proliferation without concomitant elongation), something we observed experimentally (Fig. 5c and Fig. S6f).

Turning to aphidicolin treatments, we found that it displayed an inhibition of tip volumetric growth (k_d reduced by 56%), as expected from this drug, while having a more moderate effect on tip elongation speed (v_0 reduced by 42%). Modelling these changes in the model from day 7 predicted a decrease in branch thickness (due to continued elongation with less proliferation), a feature that mirrored the data of branch thinning upon aphidicolin treatment (Fig. S6d, S6h).

III. NUMERICAL SIMULATIONS OF SPATIAL BRANCHED ORGANOID GROWTH

So far, we have used a mean-field model to fit the data, where we have averaged morphological parameters over an entire organoid, and predicted the temporal variations of these averages. However, the model described above can easily be simulated spatially.

To do this, we took the framework of branching random walks in space [8], where an organoid is represented by elongating and branching tips (resp. at constant deterministic speed v_0 and stochastic rate k_b, i.e. branching as a Poisson process), as well as the ducts they leave behind. Each tip i is represented by its coordinates (x_i, y_i) in a 2D plane and each branch j has a given width w_j and length l_j (which elongates at rate v_0 if connected to an active, unbranched tip). Branch width w_j can change either due to tip elongation (if the branch is terminal, i.e. connected to an active tip, which reduces width w_j to maintain constant branch volume upon elongation - no elongation occurs if the branch is not terminal), or due to growth (which increases width at rate $K = k_d(w_0 - w_j)$ without changing length l_j).

More specifically, given that the volume of a branch j is given by $V_j = \pi l_j (w_j/2)^2$, a tip elongation from a length $l_j(t)$ at time t to a length $l_j(t + \delta t) = l_j(t) + v_0 \delta t$ at time $t + \delta t$

obeys the conservation law $V_j(t) = V_j(t + \delta t)$, meaning that it rescales width as $w_j(t + \delta t) = \frac{w_j(t)}{\sqrt{1+v_0 \delta t / l_j(t)}}$. Similarly, growth at rate K from time t to $t + \delta t$ increases branch volume by a quantity $K V_j(t) \delta t$, so that the width is rescaled as $w_j(t + \delta t) = w_j(t)\sqrt{1 + K\delta t}$.

A. Summary of parameter estimation and simulation inputs

Overall, the parameters that we use for the spatial simulations are the same as for the continuum model (with a few additional variables that have little to no impact on the resulting dynamics), which we summary here, both showing the values that we use for these parameters in Fig. 4, but also the origin of their estimation:

- Growth parameters k_d and w_0. These have been fitted from the short-term imaging of tip elongation and growth between day 7 and 10 – from a linear fit of the data shown in Fig. 4b. Best-fit values used in the simulations: $k_d = 3.9\ d^{-1}$ and $w_0 = 25$ µm.
- Tip elongation velocity v_0. These have been estimated as the average of tip elongation speed from the short-term imaging between day 7 and day 9, which show no significant correlation with other variables such as tip width w (Fig. 5h, Fig. S5g). Values used in simulation $v_0 = 80$ µm/day.
- Branching rate k_b. Estimated from the average branch length measured between day 7 and day 9 ($l_0 \approx 150$µm), which together with an estimated elongation speed of v_0, would result in a typical branching rate of $k_b = \frac{v_0}{l_0} \approx 0.55$/day. Alternatively, we can also fit it from the increase in branch number, which leads to a best fit value of $k_b = 0.75$/day, consistent with the alternative estimation. We note that the overall quantitative dynamics of organoids is only weakly affected by this range of parameters (which controls the second phase of slower exponential growth). Value used in simulations $k_b = 0.75$/day.

- Initial conditions. At the beginning of the simulations (day 1), we considered that there is a single cell, consisting of a single branch. We initialize the simulations with a width of $w = 5\ \mu m$ and length of $l = 20\ \mu m$. At each branching event, we apply the same initial condition, considering that the nascent branch has a width of $w = 5\ \mu m$.
- Branching angle. This parameter does not impact any of the results, as tips do not interact spatially, and is thus only relevant for the visualization of the trees (Fig. 4c, 4h). We take angles of branching between mother and daughter branches of $\pi/3$.

B. Simulation output: variability in organoid branching

A typical outcome of these simulations is shown in Fig. 4c, 4h - although we show in Fig. 4h that because of the stochasticity of the branching process different runs with the exact same parameters can have very different morphometrics (which could explain the variability we find in the data in a minimal way). Note that we neglected tip termination – as we model the first phase of growth where tips elongate at a near constant velocity (see above), so that the stochasticity comes purely from the timing of stochastic branching, rather than from spatial interactions between tips as in branching and terminating random walks [8].

Importantly, when averaging over many simulations ($n = 100$, as shown in Fig. 4), we find that these recapitulate not only the temporal dynamics of branching (near-constant exponential growth in the number of branches per organoid, as predicted by a constant branching rate k_b), but also the organoid to organoid variability (assessed by standard deviation in number of branches per organoids). Indeed, this standard deviation also grew exponentially with similar values as the average, providing a non-trivial prediction of our model of stochastic branching (Fig. 4f-g). Note that this model of stochastic branching also makes the prediction, at smaller scales, that branching can happen at any time with equal probabilities, so that we expect also a broad (exponential) distribution in branch length. We tested this by measuring non-terminal

branch length across organoids at day 7 to 10 during the extension phase. Keeping in mind that branches cannot have very small lengths (<15 microns, i.e. comparable to cell size), we indeed found that branch length was very broadly distributed and consistent with an exponential distribution (Fig. S5e). This is in contrast to what would happen with a Turing instability on a simple domain [9], which would lead to periodic branching and thus highly peaked distribution – although continuous ductal growth can also cause more complex dependencies. This could be addressed in the future by measuring in further detail the relationship between branch age and length [10], although the strong non-stereotypicity that we observe both between organoids and in different regions of a given organoid suggest that intrinsic branching stochasticity is a simple explanation for the broadness of the length distribution.

C. *Simulation output: temporal dynamics in branch thickening and cell number*

When comparing the average predictions of the simulations with the analytical model, we found that both agreed well with each other. In particular, when examining the total cell number per organoid, the simulations confirm the predicted cross-over between exponential growth at rate k_d initially (Fig. S6a-d, left column) when branch thickness is small (Fig. S6a-d, right column), before a second phase where thickness plateaus and growth of cell numbers (Fig. S6a-d) still follow an exponential trend but at rate k_b. Manipulating the branching rate k_b, elongation speed v_0 and division rate k_d in the computational model by halving or multiplying by two their value compared to the measured parameters confirmed these insights (see Fig. S6a-d for such a sensitivity analysis).

In particular, the simulations agreed well with the analytical prediction that branch width should plateau to a value of $w = w_0(1 - k_b/k_d)$ (which represents a compromise between thickening via growth and thinning via branching/elongation). Using the parameters inferred above, this plateau width is predicted to be approximately 20 µm, which fits well with the data after a first

phase of gradual thickening during the first days (Fig. 4e). Conversely, when examining the average number of cells per organoid, we found similar dynamics as predicted, with a first phase of very fast growth well-fitted by the model (close to k_d) before a second phase of slower growth (dictated by k_b) – see Fig. 4d.

D. Simulation output: spatial dynamics of tip and branch morphometrics

Finally, we also predict in the simulations some spatial features (which are by definition beyond the scope of the analytical, non-spatial model). For instance, newly formed branches first undergo a phase of branch thinning due to tip elongation before containing enough cells so that proliferation dominates (and width increases) - and so that branch width plateaus to its maximal value at which proliferation is arrested. Thus, the simulation predicts a dependency of width on branch "age": branches close to the origin of the organoid are the widest and less proliferative while more recently formed branches at the periphery are thinner and more proliferative. This fits qualitatively with our observations of branching morphologies (Fig. 2c-e), and to back this up more quantitatively, we separately outputted in the simulations the width of terminal branches (which are elongating) and non-terminal branches (generation of more than one since the origin). This was binned in such two categories to be able to straightforwardly compare to data, where we separately measured at all time points the width of tip branches and the width of branches past a branch point. Interestingly, we found good agreement between data and simulations, with both quantities showing a plateau in time (as does the average width across all branches), but at very different width (Fig. 4e).

In the computational model, it was also possible to explore the effect of additional potential mechanisms. Indeed, so far we have treated each branch as separate, non-communicating entities (so that volumetric growth of a branch was only due to its proliferation). However, in particular at the initial stage where a new top just branched, and volume of a new branch is very small, it could be that fluxes occur between branch and are significant for volumetric

growth. To model this in a simple way, we performed the simulations again, but allowing for pressure driven fluxes q_{ij} to occur between two branches in contact i and j (from i to j). For simplicity, we took the simplest linear constitutive equation $q_{ij} = -\alpha(w_i - w_j)$ (i.e. $\alpha > 0$ necessary for stability, meaning that wide branches flow into thin ones, a simple assumption based on the fact that width w_i is proportional to the pressure exerted on surrounding matrix). Importantly however, when exploring different values of α, we found that these did not markedly change the dynamics, when plotting the total cell number (Fig. S6a, left) or branch thickness (Fig. S6a, right). The main change was that α "smoothed" the spatial variations with branch age (for $\alpha \rightarrow \infty$, all branches have the same thickness and the mean-field model is recovered exactly).

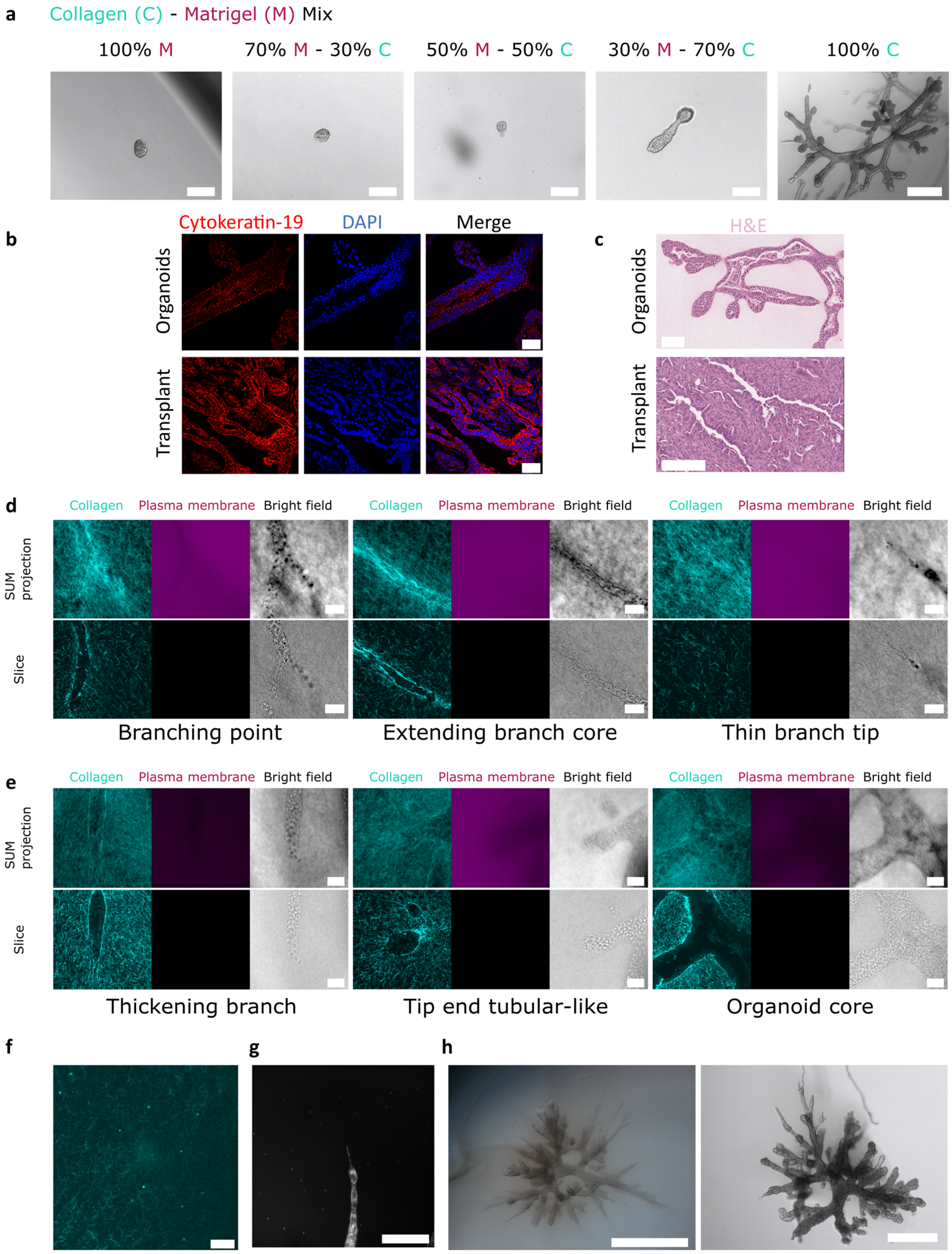

Fig. S1. Influence of the matrix composition, and remodeling of the collagen environment.

(**a**) Bright field pictures of organoids at day 13 (D13), cultured in mixtures of collagen I and Matrigel in various proportions (n = 3 individual experiments).

(**b**) Immunostaining of Cytokeratin-19 (red) and DAPI staining (blue) in D13 collagen-grown PDAC organoids and *in vivo* transplants of PDAC cells (n = 3 technical replicates).

(**c**) Haematoxylin and eosin (H&E) staining of D13 collagen-grown organoid and orthotopic transplant sections (n = 3 technical replicates).

(**d**), (**e**) Organoids were treated at D8 (d, n = 3 organoids) or D11 (e, n = 3 organoids) with Triton-X 100 and fixed. Triton-X 100 degrades the cell membrane and provoke the dissociation of organoids. Organoids are stained with CellMask, a plasma membrane marker (magenta), to ensure that the membrane has been properly washed away after the Triton-X treatment. Collagen fibers are visualized with reflection microscopy (cyan). The architecture of the collagen surrounding the organoids is preserved, indicating a plastic deformation of the ECM. Summed slices projections and single confocal slices are shown.

(**f**) Collagen fibres far away from organoids, visualized with reflection microscopy (n = 3 organoids). The fibres display no particular alignment far away from organoids. Confocal slice.

(**g**) Plasma membrane staining of an invasive protrusion at D7 (n = 13 organoids). Summed slices projection.

(**h**) Bright field pictures of a D13 organoid in collagen (left) and after collagen digestion (right) (n = 3 individual experiments).

Scale bars in (a), from left to right: 200 µm for the first four pictures, 500 µm for the fifth; in (b): 50 µm; in (c), 100 µm (top), 200 µm (bottom); (d), (e), (f): 50µm; in (g): 100 µm; in (h) from left to right: 1000 µm, 500 µm.

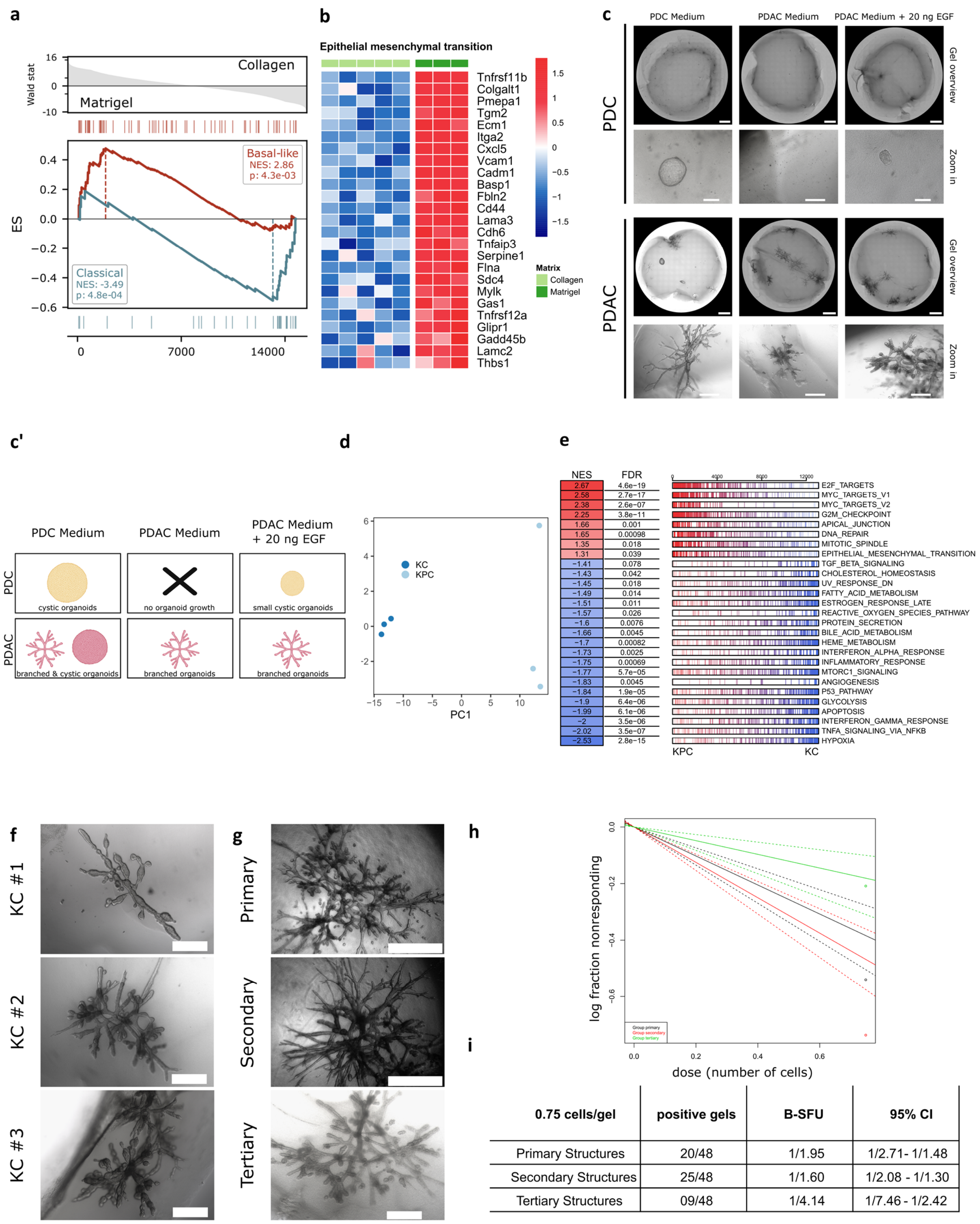

0.75 cells/gel	positive gels	B-SFU	95% CI
Primary Structures	20/48	1/1.95	1/2.71- 1/1.48
Secondary Structures	25/48	1/1.60	1/2.08 - 1/1.30
Tertiary Structures	09/48	1/4.14	1/7.46 - 1/2.42

Fig. S2. PDAC organoids epithelial plasticity drives branching morphogenesis with distinct structure forming capabilities.

(**a**) Expression scores for the “Classical” and “Basal-like” signatures displayed by Matrigel- and collagen-grown organoids at Day 13.

(**b**) Expression patterns for epithelial to mesenchymal transition (EMT) related markers, differentially expressed between Matrigel- and collagen-grown organoids at Day 13.

(**c**) Representative collagen gels containing PDC or PDAC cells, cultured in PDC medium (see Methods for the full composition) [11], DMEM + 10 % FBS (PDAC medium), and PDAC medium + 20 ng EGF. Results are summarized graphically in (**c'**): PDC form cystic organoids in PDC medium, and small cystic organoids in PDAC medium + 20 ng EGF. PDAC cells can form both branched or cystic organoids in PDC medium, and form branched organoids in PDAC medium and PDAC medium + 20 ng EGF. Created with BioRender.com

"Gel overview" pictures scale bars: 2000 µm. "Zoom-in" PDC pictures scale bars, from left to right: 200 µm, 500 µm, 100 µm. "Zoom-in" PDAC pictures scale bars: 500 µm. "Gel overview" pictures were stitched from a tile scan of a culture well and cropped to display the collagen gel.

Principal component analysis of bulk RNA sequencing of Day 13 collagen-grown organoids originating from cells derived from KC or KPC mice (**d**), and corresponding summary of gene set enrichment analysis (**e**). NES, FDR bars represent each individual gene for a given gene set.

(**f**) Representative Day 13 collagen-grown organoids originating from PDAC cells derived from three distinct KC mice (n = 3 individual experiments). Scale bars: 500 µm.

(**g**) Organoids were grown until Day 13 in collagen ("Primary" structures), dissociated at the single cell level and then seeded again in collagen, giving rise to "Secondary" structures. Repeating this process on "Secondary" structures at Day 13 in turn yielded "Tertiary" structures. Scale bars, from top to bottom: 1000 µm, 1000 µm, 500 µm.

(**h**) Log plot of non-responding well against cell dose.

(**i**) Extreme Limiting Dilution Analysis summary table.

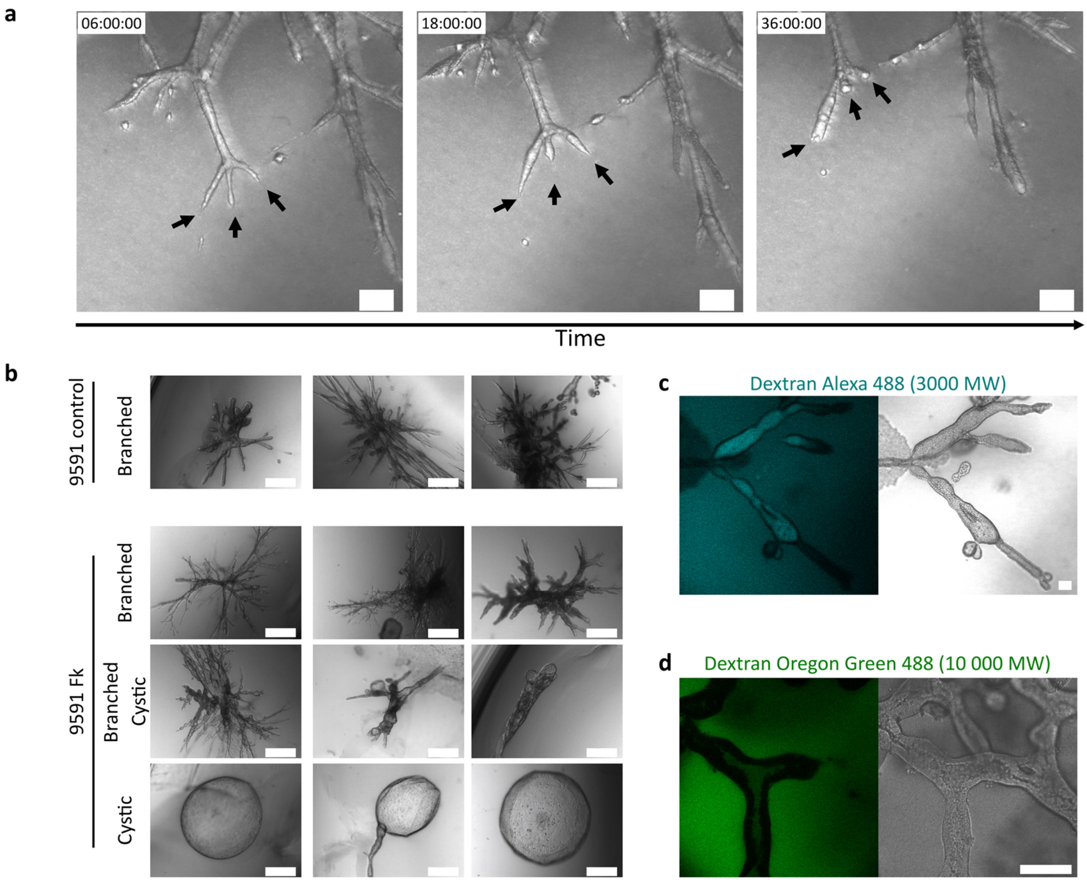

Fig. S3. Chemical perturbations via aphidicolin and forskolin.

(**a**) Bright field time-lapse of an organoid branch upon addition of 2 µg.mL^{-1} of aphidicolin at D7. Black arrows indicate spots of branch rounding and retraction.

(**b**) Bright field pictures of organoids at D13 in control conditions and upon addition of forskolin (Fk) at 10 µM. Forskolin addition triggers the apparition of cystic organoids, with lumens over-swelling (n = 3 individual experiments).

(**c**), (**d**) Dextran Alexa 488 ((c), 3000 MW, cyan, 200 µg.mL^{-1}, n = 3 replicates) or Dextran OregonGreen ((d), 10 000 MW, green, 200 µg.mL-1, n = 2 replicates) can be incorporated inside the lumen upon overnight incubation at D13-14. Confocal slices shown.

Scale bars in (a), (c), (d): 100 µm; in (b): 500 µm.

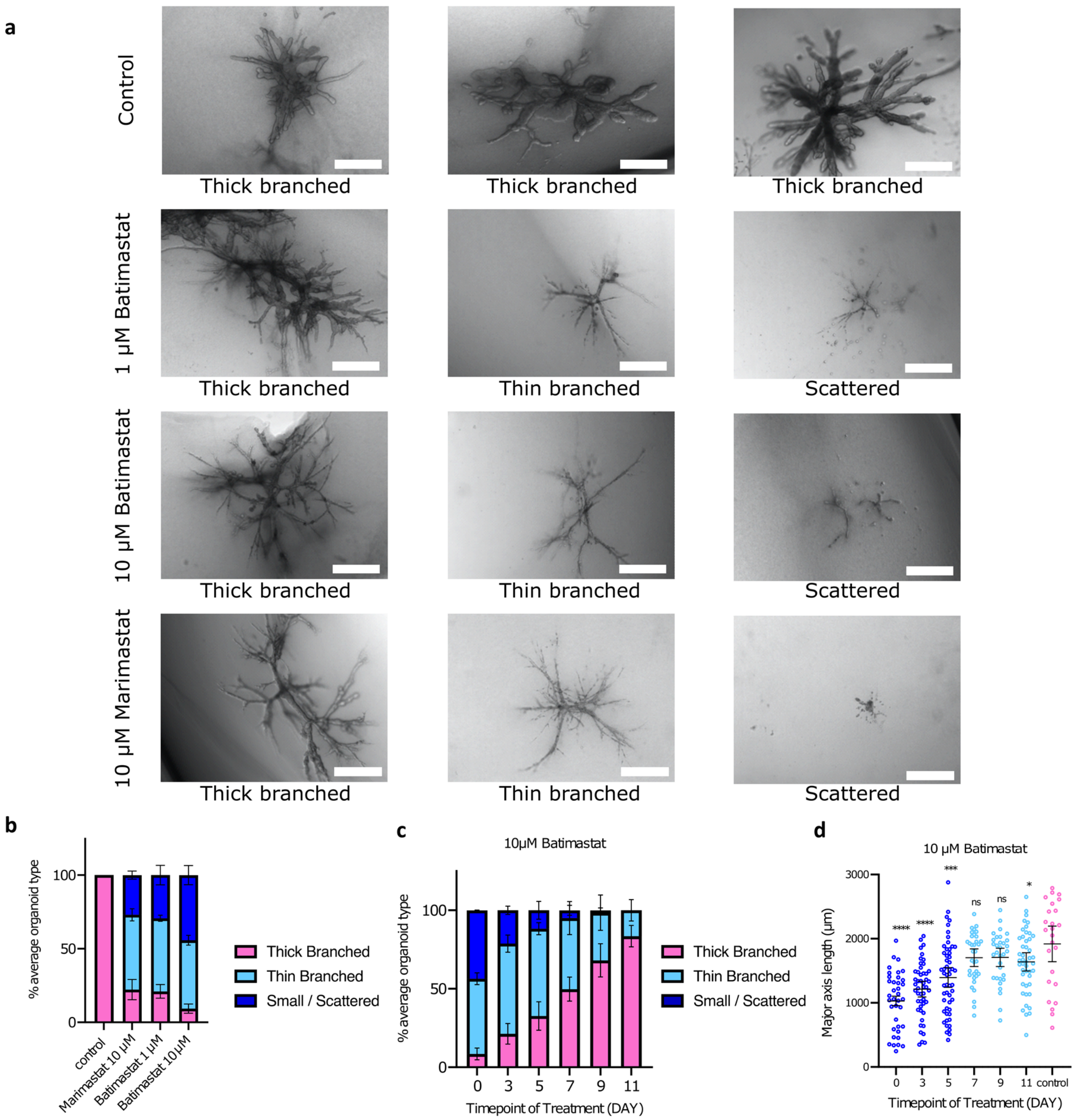

Fig. S4. Effect of MMP inhibition on collagen-grown organoid development.

(**a**) Bright field pictures of organoids at day 13 in control conditions and upon addition of batimastat at 1 µM or 10 µM, or marimastat at 10 µM (Control: n=156, Marimastat 10µM: n=106, Batimastat 1µM: n=110, Batimastat 10µM: n=129, organoids). Organoids are labelled according to their phenotype, in "Thick branched", "Thin branched" and "Scattered" categories. Scale bars: 500 µm.

(**b**) Distribution of organoid phenotypes at day 13 according to the different MMP-inhibitors used and added at seeding time (Control: n = 156, Marimastat 10µM: n = 106, Batimastat 1µM: n = 110, Batimastat 10µM: n = 129, organoids). Bars: mean ± sem.

(**c**) Distribution of organoid phenotypes at day 13 according to the addition day of 10 µM batimastat. (Control: n = 82, Batimastat: n = 589, organoids). Bars: mean ± sem.

(**d**) Major axis length of organoids at day 13 upon addition of 10 µM batimastat at different timepoints (n = 275 organoids). Bars: mean ± sem. Un-paired two tailed parametric t-test; * P = 0.0465, *** P = 0.0006, **** P ≤ 0.0001, ns P = 0.131 (batimastat addition at day 7) and P = 0.149 (batimastat addition at day 9).

All statistical tests performed against the control population.

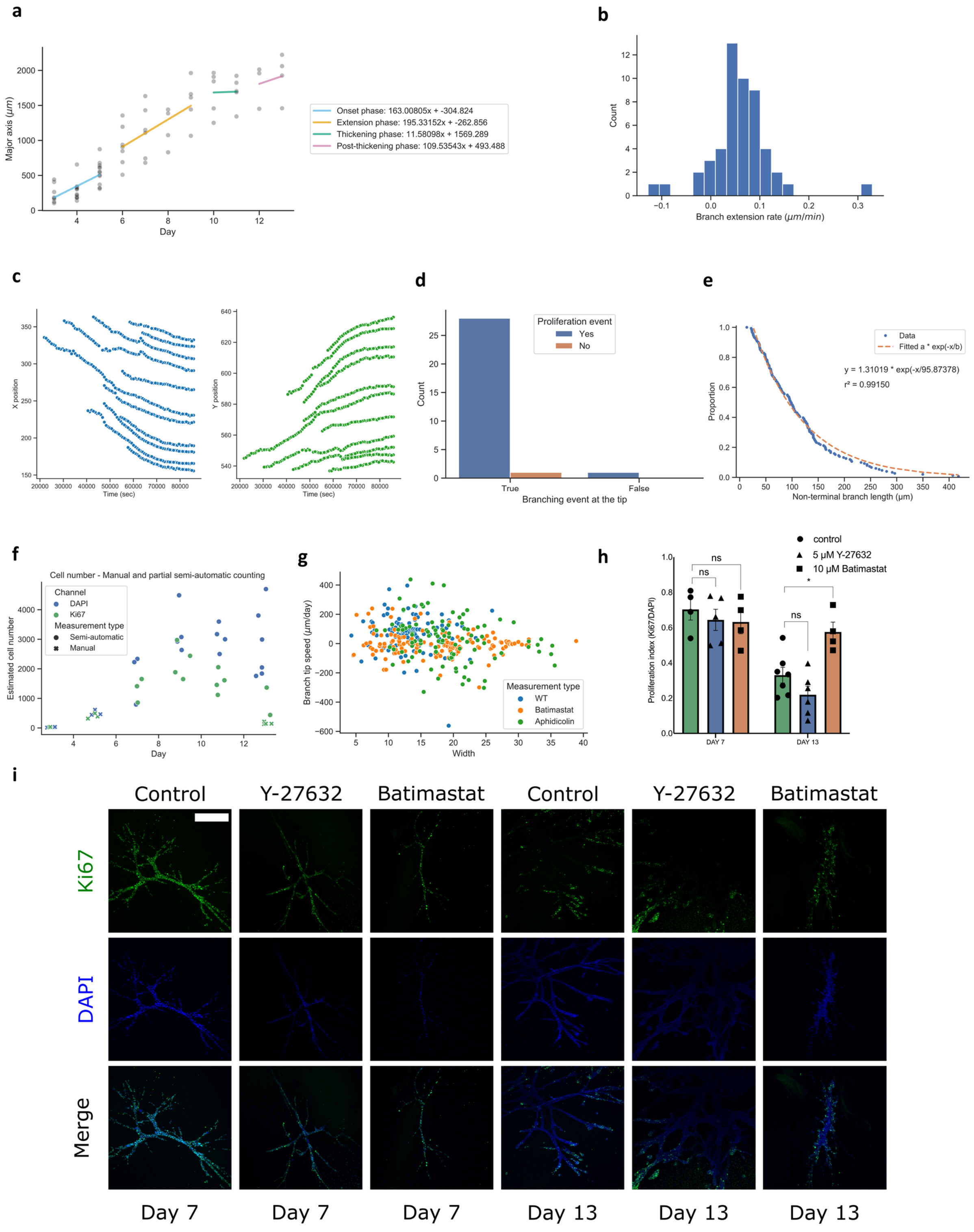

Fig. S5 Quantifications of extension and proliferation dynamics in PDAC organoids.

(**a**) Major axis length evolution of collagen-grown organoids over time with linear fits highlighting the different phases (blue: Onset, orange: Extension, green: Thickening, pink: Post-thickening; n = 71 organoids).

(**b**) Mean branch extension rate distribution between day 7 and 10 (n = 51 branches).

(**c**) X (blue) and Y (green) positions for cells in an extending branch, representative of the dominant extension behavior between day 7 and 10.

(**d**) Correlation between branching and proliferation events in the leading 6 cells of a branch tip between day 7 and 10. True-Yes: branching event was preceded by a proliferation event. True-No: branching event occurred without being preceded by a proliferation event. False-Yes: proliferation event occurred but a branching event did not follow (n = 30 events, N = 4 organoids).

(**e**) Complementary empirical cumulative distribution function of non-terminal branch lengths for organoids at day 7-10 (blue dots, n = 151 branches, N = 4 organoids) and fitted curve for an exponential distribution (dashed orange line).

(**f**) DAPI (blue) and Ki67-positive (green) cell numbers estimated from maximum projections using manual or semi-automated counting, at day 3, 5, 7, 9,11, and 13 (n = 24 organoids).

(**g**) Instantaneous branch tip speed versus branch width for untreated (WT, blue, n = 103 points, N = 3 organoids), batimastat- (orange, n = 142 points, N = 2 organoids), and aphidicolin-treated (green, n = 103 points, N = 3 organoids) organoids at the extension stage.

(**h**) Ki67-positive cells over DAPI-positive cells ratio for untreated, Y-27632-treated and batimastat-treated organoids at day 7 and day 13, and representative stainings (**i**). Bars: mean ± sem. Un-paired two-tailed parametric t test with Welch's correction; * P = 0.0116, ns P = 0.513 (day 7 control versus day 7 addition of Y-27632), P = 0.4632 (day 7 control versus day 7 addition of batimastat), P = 0.1201 (day 13 control versus day 13 addition of Y-27632). Scale bar: 100 µm. Day 7: control n = 4, batimastat n = 4, Y-27632 n = 5 organoids. Day 13: control n = 7, batimastat n = 4, Y-27632 n = 6 organoids.

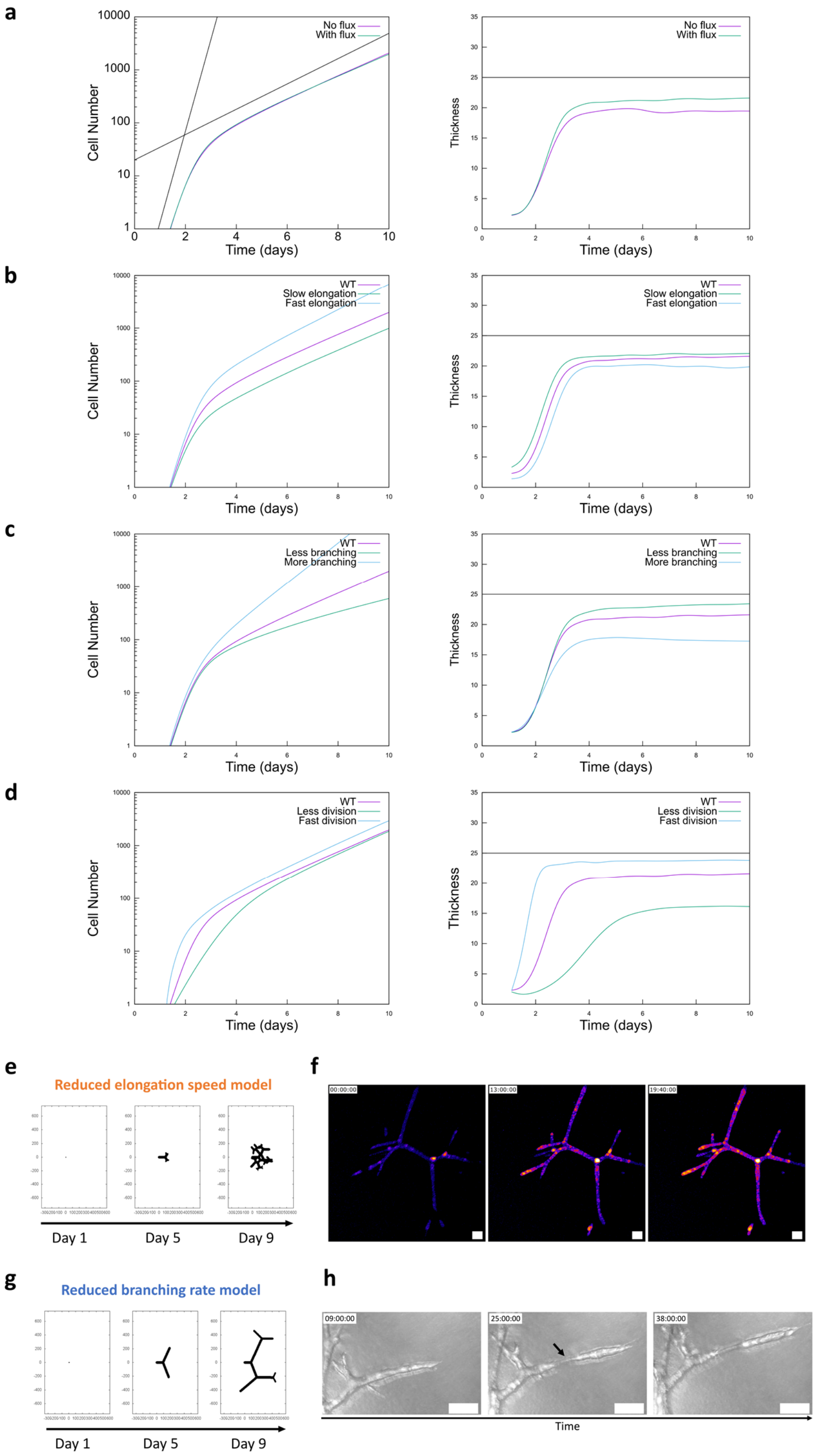

Fig. S6. Additional simulations using the minimal biophysical model.

(a-d) Model predictions of cell number evolution (left column) and of mean branch thickness evolution (right column), under different simulation parameters. Black lines in (a) are shown as a guide to the eye to indicate the initial exponential growth phase, before the plateauing.

Black lines in the right column graphs indicate the maximum width $\boldsymbol{w_0}$ allowed by the growth feedback, in absence of branching and elongation. Plots are averaged over n=30 simulations.

(**a**) With/without cell flux between branching point;

(**b**) With slower/faster elongation speeds compared to the WT parameters (elongation speed respectively divided by 2 and multiplied by 2);

(**c**) With less/more branching comparing to the WT parameters (branching rate respectively divided by 2 and multiplied by 2);

(**d**) With less/more division compared to the WT parameters (cell division rate respectively divided by 2 and multiplied by 2).

(e) Spatial simulation of the branching process over time in pancreatic organoids using a reduced branch elongation speed.

(**f**) Local thickness increase visualization in a D8-9 organoid upon addition of 10 µM batimastat. The bright spots indicate areas of increasing thickness. The organoid does not extend in size but thickens, as predicted qualitatively by the model.

(**g**) Spatial simulation of the branching process over time in pancreatic organoids using a reduced branching rate speed.

(**h**) Bright field time-lapse of an organoid branch upon addition of 2 µg.mL^{-1} of aphidicolin at D7. Black arrows indicate a spot of local branch thinning due continued cell migration but inhibited proliferation, as predicted qualitatively by the model.

Scale bars in (f), (h): 100 µm.

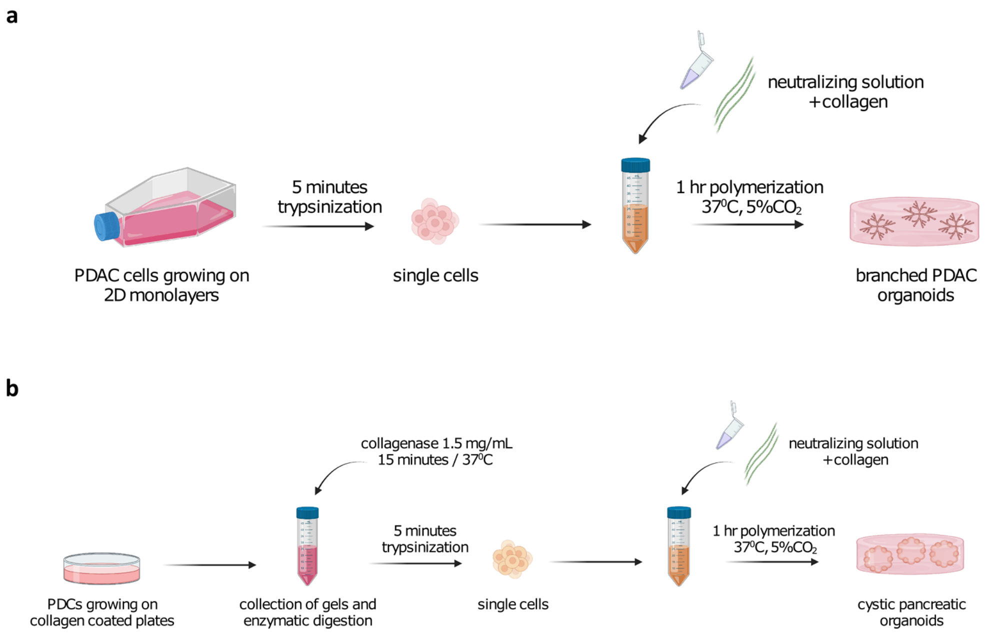

Fig. S7. Culture procedures for PDAC cells and pancreatic ductal cells.

(**a**) Schematic representation of the PDAC culture procedure.

(**b**) Schematic representation of the pancreatic ductal cells (PDC) culture procedure.

These figures were created with BioRender.com

PDC Medium composition	Final Concentration	Manufacturer	Cat. Number
DMEM-F12	NA	ThermoFisher Scientific	31330-038
D-Glucose	5mg/mL	Sigma Aldrich	G8270
ITS premix	0.5%	Corning	354350
Dexamethasone	1 µM	Sigma Aldrich	D1756
Cholera toxin	100 ng/mL	Sigma Aldrich	C8052
Penicillin/Streptomycin	1x	ThermoFisher Scientific	15140-122
Nu Serum	5%	Corning	355500
Bovine Pituitary Extract	25µg/mL	ThermoFisher Scientific	13028-014
Primocin	100 µg/mL	InvivoGen	Ant-pm-1
EGF	20ng EGF	R&D systems	2028-EG
Nicotinamide	10 mM	Sigma Aldrich	N3376

Table S1. PDC medium composition

Epitope [Clone]	Conjugation	Host	Catalogue number	Supplier	Dilution
Phalloidin	Atto-647		65906	Sigma	1:250
E-cadherin [24E10]	Alexa-488	rabbit mAb (24E10)	3199	Cell Signaling	1:50
N-cadherin [13A9]	-	mouse mAb (13A9)	14215	Cell Signaling	1:100
Krt19	-	rat	Troma III	DSHB	1:100
Ki67	-	rabbit pAb	ab15580	Abcam	1:300
α6 Integrin [GOH3]	-	rat mAb (GOH3)	Sc-19622	Santa Cruz Biotechnology	1:150
Laminin	-	rabbit pAb	L9393	Sigma	1:100
ZO-1 [ZO1-1A12]	Alexa-594	mouse mAb	339194	Invitrogen	1:100
αSMA [1A4 (asm-1)]	-	mouse mAb	MA5-11547	Thermo Fisher Scientific	1:100
Caspase 3	-	rabbit pAb	9662	Cell Signaling	1:100
Cytokeratin 7	-	rabbit mAb (EPR17078)	ab181598	Abcam	1:100

Table S2. List of primary antibodies used

Host/Isotype	Species reactivity	Conjugation	Catalogue number	Supplier	Dilution
Goat	Rat	Alexa 594	A11007	Thermo Fisher Scientific	1:250
Donkey	Rabbit	Alexa 546	A10040	Thermo Fisher Scientific	1:250
Goat	Mouse	Alexa 546	A11030	Thermo Fisher Scientific	1:250
Goat	Rabbit	Alexa 488	A11034	Thermo Fisher Scientific	1:250

Table S3. List of secondary antibodies used

References

1. Ranft, J. *et al.* Fluidization of tissues by cell division and apoptosis. *Proc. Natl. Acad. Sci.* **107**, 20863–20868 (2010).
2. Montel, F. *et al.* Stress clamp experiments on multicellular tumor spheroids. *Phys. Rev. Lett.* **107**, 1–4 (2011).
3. Delarue, M. *et al.* Compressive Stress Inhibits Proliferation in Tumor Spheroids through a Volume Limitation. *Biophys. J.* **107**, 1821–1828 (2014).
4. Delarue, M. *et al.* Self-driven jamming in growing microbial populations. *Nat. Phys.* **12**, 762–766 (2016).
5. Streichan, S. J., Hoerner, C. R., Schneidt, T., Holzer, D. & Hufnagel, L. Spatial constraints control cell proliferation in tissues. *Proc. Natl. Acad. Sci.* **111**, 5586–5591 (2014).
6. Shraiman, B. I. Mechanical feedback as a possible regulator of tissue growth. *Proc. Natl. Acad. Sci. U. S. A.* **102**, 3318–3323 (2005).
7. Basan, M., Risler, T., Joanny, J. F., Sastre-Garau, X. & Prost, J. Homeostatic competition drives tumor growth and metastasis nucleation. *HFSP J.* **3**, 265–272 (2009).
8. Hannezo, E. *et al.* A Unifying Theory of Branching Morphogenesis. *Cell* **171**, 242-255.e27 (2017).
9. Hannezo, E. & Simons, B. D. Multiscale dynamics of branching morphogenesis. *Curr. Opin. Cell Biol.* **60**, 99–105 (2019).
10. Clément, R., Douady, S. & Mauroy, B. Branching geometry induced by lung self-regulated growth. *Phys. Biol.* **9**, (2012).
11. Reichert, M. *et al.* Isolation, culture and genetic manipulation of mouse pancreatic ductal cells. *Nat. Protoc.* **8**, 1354–1365 (2013).
