# Supplementary material for: Spatiotemporal dynamics of self-organized branching in pancreas-derived organoids
Source: Nat Commun. 2022 Sep 5;13:5219. doi: 10.1038/s41467-022-32806-y (PMC9445099; doi:10.1038/s41467-022-32806-y)
Supplement: Supplementary file 3 — Description to Additional Supplementary Information [file 41467_2022_32806_MOESM3_ESM.pdf]

Description of Additional Supplementary Files

Movie S1.

3D reconstruction of a PDAC organoid staining against DAPI (blue) and Cytokeratin 7 (red) at day 13, imaged via confocal microscopy.

Movie S2.

Development of a PDAC organoid in collagen at the Onset phase, day 4- 5. Left: nuclei stained with SiRDNA, right: bright field. Both channels are summed slice projections of confocal stacks. Time-lapse imaging consists of an image taken every 15 minutes and is displayed at 20fps. Time (hh:mm:ss).

Movie S3.

Development of a PDAC organoid in collagen at the Extension phase, day 7-8. Left: nuclei stained with SiRDNA, right: bright field. Both channels are maximum projections of confocal stacks. Time-lapse imaging consists of an image taken every 13 minutes and is displayed at 20fps. Time (hh:mm:ss).

Movie S4.

Development of a PDAC organoid in collagen at the Thickening phase, day 11-13. Left: nuclei stained with SiRDNA, right: bright field. Both channels are maximum projections of confocal stacks. Time-lapse imaging consists of an image taken every 25 minutes and is displayed at 20fps. Time (hh:mm:ss).

Movie S5.

Development of a PDAC organoid in collagen at the Lumen formation phase, day 11-12. Left: nuclei

stained with SiRDNA, right: bright field. Both channels are maximum projections of confocal stacks. Timelapse imaging consists of an image taken every 10 minutes and is displayed at 20fps. Time (hh:mm:ss).

Movie S6.

Extrusion process at the Lumen formation phase, day 11-12. Bright field confocal slice. Time-lapse imaging consists of an image taken every 10 minutes and is displayed at 10fps. Time (hh:mm).

Movie S7.

Microlumen nucleation and fusion at the Lumen formation phase, day 12- 13. Bright field confocal slice. Timelapse imaging consists of an image taken every 10 minutes and is displayed at 10fps. Time (hh:mm).

Movie S8.

Deformation field at the Onset phase, day 4-5. Bright field confocal maximum projection of 10 slices, zstep=4.99 µm. Time-lapse imaging consists of an image taken every 10 minutes and is displayed at 30fps. Time (hh:mm).

Movie S9.

Deformation field at the Extension and Thickening phase, day 7-9. The switch between deformation pattern is observable around 26h15m of imaging. Bright field confocal maximum projection of 2 slices, zstep=4.99 µm. Time-lapse imaging consists of an image taken every 15 minutes and is displayed at 50fps. Time (hh:mm).

Movie S10.

Deformation field at the postThickening phase, day 13-14. Bright field confocal maximum projection of 195 slices, z-step=4.99 µm. Timelapse imaging consists of an image taken every 15 minutes and is displayed at 20fps. Time (hh:mm:ss).

Movie S11.

Thickening and retracting branch leaving behind a plasticallydeformed collagen path, day 11-13. Bright field confocal slice. Timelapse imaging consists of an image taken every 25 minutes and is displayed at 20fps. Time (hh:mm:ss).

Movie S12.

Batimastat treatment inhibits extension. Batimastat added at 10 µM from day 7 to 10. Left: nuclei stained with SiRDNA, right: bright field. Both channels are maximum projections of confocal stacks. Timelapse imaging consists of an image taken every 20 minutes and is displayed at 20fps. Time (hh:mm:ss).

Movie S13.

Aphidicolin treatment prevents proper extension. Aphidicolin added at 2 ug per mL from day 7 to 9. Left: nuclei stained with SiRDNA, right: bright field. Both channels are maximum projections of confocal stacks. Time-lapse imaging consists of an image taken every 20 minutes and is displayed at 20fps. Time (hh:mm:ss).

Movie S14.

Confocal stacks 3D reconstruction of collagen fibres, visualized with reflection microscopy, on a Day 8 organoid, treated with Triton-X 100 and fixed in paraformaldehyde. Sequentially: at a branching point, around an extending branch, at the tip of a thin branch.

Movie S15.

Confocal stacks 3D reconstruction of collagen fibres, visualized with reflection microscopy, on a Day 11 organoid, treated with Triton-X 100 and fixed in paraformaldehyde. Sequentially: at the tip of thickening branch, around a tubular-like structure at a tip end, around an organoid core.
