# Supplementary material for: Spatiotemporal dynamics of self-organized branching in pancreas-derived organoids
Source: Nat Commun. 2022 Sep 5;13:5219. doi: 10.1038/s41467-022-32806-y (PMC9445099; doi:10.1038/s41467-022-32806-y)
Supplement: Supplementary file 19 — Reporting Summary [file 41467_2022_32806_MOESM19_ESM.pdf]

nature portfolio

Corresponding author(s): Andreas R. Bausch, Maximilian Reichert

Last updated by author(s): Aug 14, 2022

Reporting Summary

Nature Portfolio wishes to improve the reproducibility of the work that we publish. This form provides structure for consistency and transparency in reporting. For further information on Nature Portfolio policies, see our Editorial Policies and the Editorial Policy Checklist.

Statistics

For all statistical analyses, confirm that the following items are present in the figure legend, table legend, main text, or Methods section.

n/a	Confirmed	
☐	☒	The exact sample size (*n*) for each experimental group/condition, given as a discrete number and unit of measurement
☐	☒	A statement on whether measurements were taken from distinct samples or whether the same sample was measured repeatedly
☐	☒	The statistical test(s) used AND whether they are one- or two-sided<br>*Only common tests should be described solely by name; describe more complex techniques in the Methods section.*
☐	☒	A description of all covariates tested
☒	☐	A description of any assumptions or corrections, such as tests of normality and adjustment for multiple comparisons
☐	☒	A full description of the statistical parameters including central tendency (e.g. means) or other basic estimates (e.g. regression coefficient) AND variation (e.g. standard deviation) or associated estimates of uncertainty (e.g. confidence intervals)
☐	☒	For null hypothesis testing, the test statistic (e.g. *F*, *t*, *r*) with confidence intervals, effect sizes, degrees of freedom and *P* value noted<br>*Give P values as exact values whenever suitable.*
☒	☐	For Bayesian analysis, information on the choice of priors and Markov chain Monte Carlo settings
☒	☐	For hierarchical and complex designs, identification of the appropriate level for tests and full reporting of outcomes
☒	☐	Estimates of effect sizes (e.g. Cohen's *d*, Pearson's *r*), indicating how they were calculated

Our web collection on statistics for biologists contains articles on many of the points above.

Software and code

Policy information about availability of computer code

Data collection	The LAS AF (version 2.6.3.8173, Leica) and LAS X (version 3.5.5.19976, Leica) softwares were used for live-cell imaging and for reflection imaging.<br>Immunostaining images were acquired using the Olympus FluoView 1200 software (Olympus Corporation). RNA library were sequenced on a NextSeq 500 (Illumina).<br>“Gel overview” images shown in Fig. S2c were acquired using the Tile Scan function of a Leica DMi8 Thunder microscope, and stitched using the “Mosaic merge” function.
Data analysis	Images were analyzed using ImageJ 1.53c or ilastik (ver 1.3.3)<br>Three-dimensional image reconstruction was performed using Imaris (8.2.0, Oxford Instrument)<br>Numerical data were analyzed using Python (ver 3.7.6) with tools from the SciPy package (ver 1.6.2), .<br>Spatial simulations were performed using Wolfram Mathematica 10.<br>Graphpad Prism (ver 9.0.2) and the R environment for statistical computing (v4.0.4) were used for statistical analysis.<br>Figures were assembled using Inkscape (ver 1.0beta2).

For manuscripts utilizing custom algorithms or software that are central to the research but not yet described in published literature, software must be made available to editors and reviewers. We strongly encourage code deposition in a community repository (e.g. GitHub). See the Nature Portfolio guidelines for submitting code & software for further information.

Data

Policy information about availability of data

All manuscripts must include a data availability statement. This statement should provide the following information, where applicable:

- Accession codes, unique identifiers, or web links for publicly available datasets
- A description of any restrictions on data availability
- For clinical datasets or third party data, please ensure that the statement adheres to our policy

Data and materials availability: Source data used to generate the graphs in this manuscript are available on a Zenodo repository with the identifier https://doi.org/10.5281/zenodo.6577226
The sequencing data that support the findings of this study are deposited in the GEO database under accession code GSE200308 [https://www.ncbi.nlm.nih.gov/geo/query/acc.cgi?acc=GSE200308].
Additional data, such as confocal stacks, that support the findings of this study are available from the corresponding authors upon reasonable request.
Code availability: Source code is available on a Zenodo repository with the identifier https://doi.org/10.5281/zenodo.6577226

Field-specific reporting

Please select the one below that is the best fit for your research. If you are not sure, read the appropriate sections before making your selection.

☒ Life sciences ☐ Behavioural & social sciences ☐ Ecological, evolutionary & environmental sciences

For a reference copy of the document with all sections, see nature.com/documents/nr-reporting-summary-flat.pdf

Life sciences study design

All studies must disclose on these points even when the disclosure is negative.

Sample size	No particular statistical method was used to define sample size.<br>The number of biological and technical replicates were chosen according to previously published experimental designs in the organoid field (Linnemann et al,2015; Buchmann, Engelbrecht et al. 2021)<br>For chemical perturbations and immunostaining experiments, a minimum of 3 independent experiments were performed.<br>For RNA-sequencing, 3-5 technical replicates were performed.<br>For live imaging morphometrics quantification 2-3 replicates were measured for each set of experiment, and experiments were performed at least twice with similar outcomes.<br>The detailed sample sizes are specified in figure captions for each experiment.
Data exclusions	For live imaging experiments, organoids were visually assessed for morphology and viability.
Replication	Experiments were reliably reproduced. Experiments were performed on different days with different batches of organoids, and sample sizes are reported in figure captions for each experiment.
Randomization	After visually assessing their morphology and viability, organoids were randomly allocated into the experimental groups and parameters for different experiments were measured at random.
Blinding	Authors were not blinded during experiments and analysis, as data analysis is based on quantitative measurements after morphology and viability assessment, without further data exclusion.

Reporting for specific materials, systems and methods

We require information from authors about some types of materials, experimental systems and methods used in many studies. Here, indicate whether each material, system or method listed is relevant to your study. If you are not sure if a list item applies to your research, read the appropriate section before selecting a response.

Materials & experimental systems

n/a	Involved in the study	
☐	☒	Antibodies
☐	☒	Eukaryotic cell lines
☒	☐	Palaeontology and archaeology
☐	☒	Animals and other organisms
☒	☐	Human research participants
☒	☐	Clinical data
☒	☐	Dual use research of concern

Methods

n/a	Involved in the study	
☒	☐	ChIP-seq
☒	☐	Flow cytometry
☒	☐	MRI-based neuroimaging

Antibodies

Antibodies used	Also stated in the Supplementary Materials section under Table 2 and 3.<br><br>Primary antibodies (Catalog number) [Dilution]:<br>Atto-647 Phalloidin, (65906, Sigma), [1:250]<br>anti-E-cadherin Alexa 488, rabbit mAb (24E10), (3199, Cell Signaling), [1:50]<br>anti-N-cadherin, mouse mAb (13A9), (14215, Cell Signaling), [1:100]<br>anti-Krt19, rat, (Troma III, DSHB), [1:100]<br>anti-Ki67, rabbit pAb, (ab15580, Abcam), [1:300]<br>anti-α6 Integrin, rat mAb (GOH3), (Sc-19622, Santa Cruz Biotechnology), [1:150]<br>anti-laminin, rabbit pAb, (L9393, Sigma), [1:100]<br>anti-ZO-1 Alexa 594, mouse mAb (ZO1-1A12), (339194, Invitrogen), [1:100]<br>anti-αSMA, mouse mAb (1A4 (asm-1)) , (MA5-11547, Thermo Fisher Scientific), [1:100]<br>anti-Caspase 3, rabbit pAb, (9662, Cell Signaling), [1:100]<br>anti-Cytokeratin 7, rabbit mAb (EPR17078), (ab181598, Abcam) [1:100]<br><br>Secondary antibodies (Catalog number) [Dilution] :<br><br>Goat anti-Rat Alexa 594, (A11007, Thermo Fisher Scientific), [1:250]<br>Donkey anti-Rabbit Alexa 546, (A10040, Thermo Fisher Scientific), [1:250]<br>Goat anti-Mouse Alexa 546, (A11030, Thermo Fisher Scientific), [1:250]<br>Goat anti-Rabbit Alexa 488, (A11034, Thermo Fisher Scientific), [1:250]
Validation	Validation statements available from manufacturers:<br><br>Primary antibodies:<br><br>Atto-467 Phalloidin (https://www.sigmaaldrich.com/catalog/product/sigma/65906?lang=de&region=DE)<br>anti-E-cadherin Alexa 488 (https://www.cellsignal.com/products/antibody-conjugates/e-cadherin-24e10-rabbit-mab-alexa-fluor-488-conjugate/3199)<br>anti-N-cadherin (https://www.cellsignal.com/products/primary-antibodies/n-cadherin-13a9-mouse-mab/14215)<br>anti-Krt19 (https://dshb.biology.uiowa.edu/TROMA-III)<br>anti-Ki67 (https://www.abcam.com/Ki67-antibody-ab15580.html)<br>anti-α6 Integrin (https://www.scbt.com/p/integrin-alpha6-antibody-goh3)<br>anti-laminin (https://www.sigmaaldrich.com/catalog/product/sigma/l9393?lang=de&region=DE)<br>anti-ZO-1 Alexa 594 (https://www.thermofisher.com/antibody/product/ZO-1-Antibody-clone-ZO1-1A12-Monoclonal/339194)<br>anti-αSMA (https://www.thermofisher.com/antibody/product/Alpha-Smooth-Muscle-Actin-Antibody-clone-1A4-asm-1-Monoclonal/MA5-11547)<br>anti-Caspase 3 (https://www.cellsignal.com/products/primary-antibodies/caspase-3-antibody/9662)<br>anti-Cytokeratin 7 (https://www.abcam.com/cytokeratin-7-antibody-epr17078-cytoskeleton-marker-ab181598.html)<br><br>Secondary antibodies:<br><br>Goat anti-Rat Alexa 594 (https://www.thermofisher.com/antibody/product/Goat-anti-Rat-IgG-H-L-Cross-Adsorbed-Secondary-Antibody-Polyclonal/A-11007)<br>Donkey anti-Rabbit Alexa 546 (https://www.thermofisher.com/antibody/product/Donkey-anti-Rabbit-IgG-H-L-Highly-Cross-Adsorbed-Secondary-Antibody-Polyclonal/A10040)<br>Goat anti-Mouse Alexa 546 (https://www.thermofisher.com/antibody/product/Goat-anti-Mouse-IgG-H-L-Highly-Cross-Adsorbed-Secondary-Antibody-Polyclonal/A-11030)<br>Goat anti-Rabbit Alexa 488 (https://www.thermofisher.com/antibody/product/Goat-anti-Rabbit-IgG-H-L-Highly-Cross-Adsorbed-Secondary-Antibody-Polyclonal/A-11034)

Eukaryotic cell lines

Policy information about cell lines

Cell line source(s)	Primary tumour cells were collected from a genetically engineered mouse model of pancreatic cancer Ptf1aCre/+; KrasG12D/+ (KC mice) and from a Pdx1Cre/+; KrasG12D/+; TP53 fl/fl mouse (KPC mouse)
Authentication	Cells have been authenticated in the laboratory from which the cells were received.
Mycoplasma contamination	Cell lines were tested negative for mycoplasma contamination.
Commonly misidentified lines (See ICLAC register)	The cell lines used in this study are not present in the ICLAC register.

Animals and other organisms

Policy information about studies involving animals; ARRIVE guidelines recommended for reporting animal research

Laboratory animals	Primary tumour cells were collected from a genetically engineered mouse model of pancreatic cancer Ptf1aCre/+; KrasG12D/+ (KC mice) and from a Pdx1Cre/+; KrasG12D/+; TP53 fl/fl mouse (KPC mouse). For the endogenous mouse model (Mueller et al. 2018),

mice were maintained on C57Bl/6;129S6/SvEv mixed background, and female and male mice were randomly submitted to respective tumour cohorts. For the generation of double-mutants, pancreas-specific Cre lines were intercrossed with KrasG12D-Panc (PK mice). For the orthotopic transplantation female athymic nude mice, aged between 7 to 9 weeks, with NU(NCr)-Foxn1nu background (Charles River) were used.

Wild animals	Wild animals were not used in this study.
Field-collected samples	This study did not involve field-collected samples.
Ethics oversight	For the endogenous mouse model (Mueller et al. 2018) as well as the orthotopic transplantation model, mice were euthanized in compliance with the European guidelines for the care and use of laboratory animals. In detail, animals were euthanized when a palpable abdominal mass above 1.5 cm, ascites, signs of sickness or a weight loss of more than 15% of body weight. Mice were monitored on a daily basis regarding general health status as well as body weight and housed under specific-pathogen-free conditions. Animal studies were approved by the Institutional Animal Care and Use Committees of Technische Universität München (Regierung von Oberbayern, Munich, Germany).

Note that full information on the approval of the study protocol must also be provided in the manuscript.
